# Molecular Characterization of GABA-A Receptor Subunit Diversity within Major Peripheral Organs and Their Plasticity in Response to Early Life Psychosocial Stress

**DOI:** 10.3389/fnmol.2018.00018

**Published:** 2018-02-06

**Authors:** Ethan A. Everington, Adina G. Gibbard, Jerome D. Swinny, Mohsen Seifi

**Affiliations:** Institute for Biomedical and Biomolecular Sciences and School of Pharmacy and Biomedical Sciences, University of Portsmouth, Portsmouth, United Kingdom

**Keywords:** chloride transporters, GABA, KCC2, peripheral nervous system, psychosocial stress, NKCC1/2

## Abstract

Gamma aminobutyric acid (GABA) subtype A receptors (GABA_A_Rs) are integral membrane ion channels composed of five individual proteins or subunits. Up to 19 different GABA_A_R subunits (α1–6, β1–3, γ1–3, δ, ε, θ, π, and ρ1–3) have been identified, resulting in anatomically, physiologically, and pharmacologically distinct multiple receptor subtypes, and therefore GABA-mediated inhibition, across the central nervous system (CNS). Additionally, GABA_A_R-modulating drugs are important tools in clinical medicine, although their use is limited by adverse effects. While significant advances have been made in terms of characterizing the GABA_A_R system within the brain, relatively less is known about the molecular phenotypes within the peripheral nervous system of major organ systems. This represents a potentially missed therapeutic opportunity in terms of utilizing or repurposing clinically available GABA_A_R drugs, as well as promising research compounds discarded due to their poor CNS penetrance, for the treatment of peripheral disorders. In addition, a broader understanding of the peripheral GABA_A_R subtype repertoires will contribute to the design of therapies which minimize peripheral side-effects when treating CNS disorders. We have recently provided a high resolution molecular and function characterization of the GABA_A_Rs within the enteric nervous system of the mouse colon. In this study, the aim was to determine the constituent GABA_A_R subunit expression profiles of the mouse bladder, heart, liver, kidney, lung, and stomach, using reverse transcription polymerase chain reaction and western blotting with brain as control. The data indicate that while some subunits are expressed widely across various organs (α3–5), others are restricted to individual organs (γ2, only stomach). Furthermore, we demonstrate complex organ-specific developmental expression plasticity of the transporters which determine the chloride gradient within cells, and therefore whether GABA_A_R activation has a depolarizing or hyperpolarizing effect. Finally, we demonstrate that prior exposure to early life psychosocial stress induces significant changes in peripheral GABA_A_R subunit expression and chloride transporters, in an organ- and subunit-specific manner. Collectively, the data demonstrate the molecular diversity of the peripheral GABA_A_R system and how this changes dynamically in response to life experience. This provides a molecular platform for functional analyses of the GABA–GABA_A_R system in health, and in diseases affecting various peripheral organs.

## Introduction

The neurotransmitter gamma aminobutyric acid (GABA) is capable of mediating a rich variety of cellular communication patterns, throughout the entire nervous system, by engaging a multitude of molecularly and functionally diverse GABA receptor subtypes (Avoli and Krnjevic, 2016). One such major class of GABA receptors are GABA-A receptors (GABA_A_Rs), which are principally engaged in mediating the rapid effects of GABA. GABA_A_Rs are composed of individual proteins, called subunits, which assemble in a heteropentameric structure to form an anion-permeable ion channel. Although only five subunits are required to form a functional receptor complex, 19 molecularly distinct subunits have so far been identified; these are classified as α1–6, β1–3, γ1–3, δ, ε, θ, π, and ρ1–3. As a result, GABA_A_Rs, composed of various subunit combinations, give rise to numerous receptor subtypes. Characterizing the different GABA_A_R subtypes, within all the various branches of the nervous system, is essential for determining the contribution of the body’s GABA–GABA_A_R system, in health and disease.

Within the brain, GABA_A_Rs are one of the most comprehensively studied classes of neurotransmitter receptors. Combinatorial evidence over the last ∼30 years has revealed that within the central nervous system (CNS), molecularly distinct GABA_A_R subunits are diverse according to their cellular and subcellular expression patterns (Fritschy and Panzanelli, 2014), their activation and deactivation kinetics (Farrant and Nusser, 2005), and their gating by different pharmacological ligands (Rudolph and Knoflach, 2011). The overall effect of GABA_A_R activation on cellular excitability is dependent on the chloride ion gradient across the cell membrane, which is maintained by the potassium-chloride transporter member 5 (KCC2), the Na–K–Cl co-transporter 1 (NKCC1) and Na–K–Cl co-transporter 2 (NKCC2) (Payne et al., 2003; Chamma et al., 2012). Although GABA is the principal inhibitory neurotransmitter within the adult CNS, it initially has an excitatory function in the early postnatal rodent brain (Ben-Ari et al., 1989, 2012). This is due in part to the developmental upregulation in the expression of KCC2 (Rivera et al., 2005). Accordingly, there is a negative shift in the reversal potential for chloride ions with brain maturation. These brain GABA_A_R profiles are also sensitive to psychosocial stress (Caldji et al., 2004; Corteen et al., 2015), suggesting a class of molecules which change dynamically with life experience. As a result, we have a considerable understanding of the contributions of various GABA_A_R subtypes to native brain function and various brain disorders. This understanding has contributed to the development of numerous valuable GABA_A_R-modulating drugs in clinical medicine, primarily for disorders of CNS origin. However, considerable less attention has been paid to the peripheral nervous system (PNS) GABA_A_R system, despite evidence of its expression within various organ system, thus limiting the possible exploitation of GABA_A_R-modulating drugs for any associated disorders.

GABA is expressed within various types of peripheral tissue of both rodents and humans, such as pancreatic islet cells, the oviduct, the gastrointestinal tract (GIT) and adrenal chromaffin cells (Tanaka, 1985). GABA has been demonstrated to function as a neurotransmitter within various organ systems such as lung (Yabumoto et al., 2008) and the kidney (Sasaki et al., 2006). Converging evidence points to GABA_A_R subunit diversity explored across peripheral tissues (Akinci and Schofield, 1999) and in different species such as rat (Akinci and Schofield, 1999; Jin et al., 2008), mouse (Tyagi et al., 2007), and human (Mizuta et al., 2008; Zhang et al., 2013). Inextricably linked to GABA_A_R function are the ion transporters that maintain the gradients for the chloride ions which permeate such ion channels. Indeed, in contrast to the brain, GABAergic neurotransmission within peripheral branches of the nervous system, such as the enteric nervous system (ENS) of the GIT, have been shown to be excitatory in adulthood (Cherubini and North, 1984; Krantis, 2000). This is due to peripheral excitatory action of GABA within the ENS because of the lack of the KCC2 transporter in adulthood (Gameiro et al., 2005; Xue et al., 2009).

We have recently provided the first high resolution molecular and function characterization of the GABA_A_Rs within the ENS of the mouse colon (Seifi et al., 2014). However, the expression patterns of these receptors within major peripheral organs such as the lung, heart, liver, kidney, and bladder still remain unclear. Furthermore, the developmental expression levels of the chloride transporters that determine the effect of GABA_A_Rs on cellular excitability, in other peripheral organs, remains unclear. In this study, we provide the first comprehensive characterization of GABA_A_R subunits expression within such vital peripheral organs. We also determine the developmental changes in the chloride transporters mRNA expression within various peripheral organs. Finally, we demonstrate that experience of prior early life stress (ELS) induces significant changes in the expression of specific GABA_A_R subunits and chloride ion transporter in an organ-specific manner, in adulthood.

## Materials and Methods

All procedures involving animal experiments were approved by the Animal Welfare and Ethical Review Body of the University of Portsmouth and were performed by a personal license holder, under a Home Office-issued project license, in accordance with the Animals (Scientific Procedures) Act, 1986 (United Kingdom) and associated procedures.

### Reverse Transcription Polymerase Chain Reaction

The first aim of this study was to determine which particular GABA_A_R subunits are expressed at the mRNA levels within various peripheral organs of the mouse. Therefore, reverse transcription polymerase chain reaction (RT-PCR) was performed on freshly isolated pieces of the mouse lung, heart, liver, stomach, kidney, and bladder with matched brain tissue used as positive control since all the GABA_A_R subunits investigated have been shown to be expressed within the mouse brain.

Adult male C57BL/6J mice (Charles River Laboratories; *N* = 3) were killed by cervical dislocation and segments of the lung, heart, liver, stomach, kidney, bladder, and whole brain were removed and snap frozen in liquid nitrogen and stored at -80°C until used. The frozen tissue was then homogenized in appropriate amounts of lysis buffer from which RNA was extracted using an RNeasy mini kit (Qiagen, 74104) according to the manufacturer’s protocol. The quality and quantity of the extracted RNA in each tissue was examined with spectrophotometry (Thermo Scientific^TM^ NanoDrop^TM^).

The reverse transcription and PCR were performed according to our previously published protocols (Seifi et al., 2014). Equal amounts of RNA from each tissue was reverse-transcribed into first-stand cDNA in the following reaction: 2 μl of 10× M-MuLV Reverse Transcriptase Reaction Buffer which included, in the final concentration, 50 mM Tris–HCl, 10 mM DTT, 75 mM KCl and 3 mM MgCl2 (BioLabs), 0.1 mM Oligo(dT)18 Primer (Thermo Fisher Scientific), 1 mM dNTP Mix (Thermo Fisher Scientific), 0.5 μl of M-Mulv reverse transcriptase (Applied Biosystems), and 0.5 μl of riboLock (Thermo Fisher Scientific). Each reaction was then incubated at 37°C for 2 h. Equal amounts of cDNA (1–2 μl) were then used for subsequent PCR using GoTaq Green Master Mix (Promega), PCR grade water and specific primers for 16 different GABA_A_R subunit. Exon–exon spanning GABA_A_R subunit specific PCR primers used in the study (**Table 1**) have been previously published (Glassmeier et al., 1998; Gustincich et al., 1999; Tan K.R. et al., 2011). The RT-PCR transcript products for the GABA_A_R subunits from all investigated tissues were run on a 2% agarose gel and the DNA was visualized under ultraviolet light using a SYBR green-based DNA stain.

**Table 1:** Details of RT-PCR primer sequences used in the study.

Subunit	Primer sequence	RT-PCR product length (bp)	Reference
GABA_A_R α1	CCA AGT CTC CTT CTG GCT CAA CA	111	Tan S. et al. (2011)
	GGG AGG GAA TTT CTG GCA CTG AT		
GABA_A_R α2	TTA CAG TCC AAG CCG AAT GTC CC	103	Tan S. et al. (2011)
	ACT TCT GAG GTT GTG TAA GCG TAG C		
GABA_A_R α3	CAA GAA CCT GGG GAC TTT GTG AA	119	Tan S. et al. (2011)
	AGC CGA TCC AAG ATT CTA GTG AA		
GABA_A_R α4	GAG ACT GGT GGA TTT TCC TAT GG	94	Tan S. et al. (2011)
	GGT CCA GGT GTA GAT CAT CTC ACT		
GABA_A_R α5	CCC TCC TTG TCT TCT GTA TTT CC	99	Tan S. et al. (2011)
	TGA TGT TGT CAT TGG TCT CGT CT		
GABA_A_R α6	TAC AAA GGA AGA TGG GCT ATT	439	Glassmeier et al. (1998)
	ACG ATG GGC AAA GTC AGA GAG		
GABA_A_R β1	GGG GCT TCT CTC TTT TCC CGT GA	334	Gustincich et al. (1999)
	GGT GTC TGG TAC CCA GAG TTG GT		
GABA_A_R β2	CAA CTC TGG GTG CCT GAC ACC TA	495	Gustincich et al. (1999)
	TCC TAA TGC AAC CCG TGC AGC AG		
GABA_A_R β3	GGT TTG CTG CGC TCA GAG CGT AA	390	Gustincich et al. (1999)
	TAC AGC ACT GTC CCA TCA GGG T		
GABA_A_R γ1	CAG TTT GCA TTT GTA GGG TTA CG	165	Gustincich et al. (1999)
	AGA CAC CCA GGA AAG AAC CAC TG		
GABA_A_R γ2	GGT GGA GTA TGG CAC CCT GCA TT	322	Gustincich et al. (1999)
	AGG CGG TAG GGA AGA AGA TCC GA		
GABA_A_R γ3	TGC TCG GTC CAG GAG GGT AGA	592	Gustincich et al. (1999)
	CTG ATC AGC TGC CTC AAC TGA ATT TTT		
GABA_A_R δ	GAC TAC GTG GGC TCC AAC CTG GA	398	Gustincich et al. (1999)
	ACT GTG GAG GTG ATG CGG ATG CT		
GABA_A_R ε	CAA TGC GAA GAA CAC TTG GAA GC	225	Gustincich et al. (1999)
	CTG GCA GCA GCA GCT TCT ATC TT		


### Western Blotting

Adult male C57BL/6J mice (Charles River Laboratories; *N* = 3) were killed by cervical dislocation and segments of the lung, heart, liver, stomach, kidney, bladder, and whole brain were removed and snap frozen in liquid nitrogen and stored at -80°C until used. The tissue was then homogenized and lysed in ice-cold RIPA lysis buffer [25 mM Tris–HCl (pH 7.6), 150 mM NaCl, 1% NP-40, 1% sodium deoxycholate, 0.1% sodium dodecyl sulfate] supplemented with a cocktail of protease and phosphatase inhibitors (Thermo Fisher Scientific). Subsequently, the tissue lysate was clarified by centrifugation of samples at 4°C.

The extracted proteins were then boiled at 95°C for 3 min and separated by 10% sodium dodecyl sulfate polyacrylamide gel electrophoresis. The gels were then placed in a wet transfer tank and the separated proteins were electrotransferred onto an activated polyvinylidene fluoride membrane (Thermo Fisher Scientific). The membranes were then incubated on a shaker for 1 h at room temperature with a blocking solution containing Tris-buffered saline-Tween 0.1% (TBS-Tween; Thermo Fisher Scientific) and 3% non-fat dry milk. After the blocking stage, the membranes were incubated with primary antibodies diluted in the blocking solution over night at 4°C. The next day, membranes were washed four times (5 min each time) with TBS-Tween solution in order to wash the excess primary antibodies off the membranes. Subsequently, membranes were incubated with appropriate horseradish peroxidase (HRP)-conjugated secondary antibody diluted in the blocking solutions for 2 h. The membranes were then washed in TBS-Tween (4 × 5 min) and incubated with an enhanced chemiluminescence development reagent (Luminata Forte; Millipore) for 3 min and visualized with a high sensitivity CCD camera imaging platform (ChemiDoc MP; Bio-Rad, Hemel Hempstead, United Kingdom).

The primary antibodies used were: mouse anti GABA_A_R α1, 1:500 (NeuroMab clone N95/35); rabbit anti GABA_A_R α2, 1:1000 (Millipore, catalog number AB5948); rabbit anti GABA_A_R α3, 1:1000 (Synaptic Systems, catalog number 224 303); mouse anti GABA_A_R α4, 1:500 (NeuroMab, clone N398A/34); rabbit anti GABA_A_R α5 (Synaptic Systems, catalog number 224 503), rabbit anti GABA_A_R γ2, 1:1000 (Synaptic Systems, catalog number 224 003); mouse anti β-actin, 1:1000 (Cell Signaling, catalog number 3700). Secondary antibodies used were donkey anti-rabbit HRP (Promega, Southampton, United Kingdom) and anti-mouse HRP (1:5000) (Promega, Southampton, United Kingdom).

### ELS Paradigm

Within the CNS, the expression of the GABA_A_R system is highly dynamic and changes in response to individual stimuli such as stress (Caldji et al., 2003; Hsu et al., 2003; Verkuyl et al., 2004; Maguire and Mody, 2007; Zheng et al., 2007; Corteen et al., 2015). We therefore investigated whether prior ELS results in altered GABA_A_R expression within peripheral organs, focusing on the GABA_A_R α1–5 and γ2 subunits. For these experiments, we exploited a validated animal model of ELS which is based on a fragmented mother–pup interaction during the first week of life (Rice et al., 2008; Gunn et al., 2013).

Briefly, pregnant dams were housed together with male partners and monitored every 12 h for the birth of pups. The day of birth was termed postnatal day 0 (PND 0). Both the control and ELS dams were left undisturbed until PND 2. On PND 2, litters were adjusted to a maximum of eight pups. Only male offspring were used for analyses. Control dams were housed in standard sawdust bedding and provided with sufficient nesting material (1 square; Nestlets^R^, Ancare). In the ELS cages, dams were provided with reduced nesting material (2/3 of a square) placed upon a raised, fine-gauge (5 mm) steel mesh platform. The cage floor was covered with a small amount of sawdust to prevent ammonia build-up. All litters were left undisturbed between PND 2 and 9. On PND 9, both control and ELS pups were returned with the dams to cages with standard bedding and nesting material. Offspring remained with the dams until weaning at PND 22–23. Once the animal reached adulthood (PND 90), the effect of ELS on the expression of various GABA_A_R subunits in adulthood was assessed within peripheral organs, using quantitative RT-PCR.

### Quantitative Real-Time Polymerase Chain Reaction

Adult male mice from control and ELS groups were killed by cervical dislocation and tissue homogenates of whole brain, lung, heart, liver, stomach, kidney, and bladder prepared. RNA was extracted from the samples and then reverse transcribed into cDNA as described earlier.

Quantitative PCR (qPCR) amplification was performed in 96-well plates in a master mix for probes (Roche, Burgess Hill, United Kingdom) and run on a LightCycler^®^ 96 System (Roche). The qPCR amplifications for the mouse *Gabra1* (assay ID: Mm00439046_m1), *Gabra2* (assay ID: Mm00433435_m1), *Gabra3* (assay ID: Mm01294271_m1), *Gabra4* (assay ID: Mm00802631_m1), *Gabra5* (assay ID: Mm00621092_m1), *Gabrg2* (assay ID: Mm00433489_m1) genes were performed using pre-designed TaqMan primers/probes purchased from Life Technologies (Thermo Fisher scientific). *Gapdh* (assay ID: Mm99999915_g1) gene expression was used as the housekeeping gene in every reaction. The qPCR cycling conditions entailed 95°C for 10 min and 40 cycles of 95°C for 15 s and 60°C for 60 s (LightCycler^®^ 96 System, Roche). Standard curves were generated for Gabra1–5 and Gabrg2 using serial dilutions of a known amount of mRNA extracted from each organ which were then reverse transcribed into cDNA. Each measurement was performed in duplicate and each Ct value was then converted into ng mRNA using linear regression analysis of the standard curve (Microsoft Excel). Each ng mRNA value was then normalized against the ng housekeeping gene level within the same sample and the mean mRNA levels for every sample was finally calculated and compared across all experimental groups.

In another set of experiments, using normally reared mice, we quantified the developmental mRNA expression levels of the main membrane transporters that determine chloride gradients across cell membranes, namely the KCC2, the NKCC1 and NKCC2, within the different organs, with brain used as a control. Expression levels, using qPCR, were compared between animals aged PND 6 and 60, since developmental changes are known to occur within this time window (Payne et al., 2003; Ben-Ari et al., 2012). The following pre-designed TaqMan primers/probes were used: *Slc12a5* (assay ID: Mm00803929_m1), *Slc12A2* (assay ID: Mm01265951_m1) and *Slc12A1* (assay ID: Mm01275821_m1) genes. *Gapdh* was used as the housekeeping gene (Thermo Fisher Scientific).

### Statistical Analysis

All statistical analyses were performed using GraphPad Prism 7 (GraphPad Inc, La Jolla, CA, United States). Animals were randomly assigned to treatment groups. All results are expressed as mean ± SEM. Statistical comparisons between different animal groups and treatments were assessed using the unpaired Student’s *t*-test. A *P*-value less than 0.05 was considered statistically significant.

## Results

### GABA_A_R Subunit mRNA Expression within Specific Peripheral Organs

**FIGURE 1:**
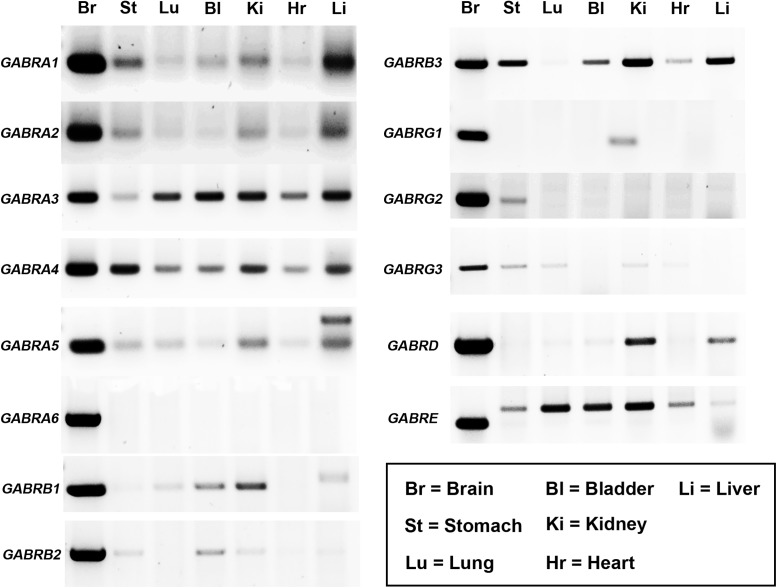
Diversity of GABA_A_R subunit expression within various organs of the mouse. Representative gel electrophoresis images of mRNA transcripts for various GABA_A_R subunits using RT-PCR and homogenates from the whole brain (Br), stomach (St), lung (Lu), bladder (Bl), kidney (Ki), heart (Hr), and liver (Li) obtained from adult male C57BL/6 mice. Corresponding amplicons of the same size bands to those obtained from the brain were consistently detected for various GABA_A_R subunits within a number of peripheral organs. *N* = 3 animals.

Previous studies have reported the expression of up to 19 different GABA_A_R subunits within the rodent CNS (Sieghart, 2006). However, the mammalian gene expression of these receptor subunits within distinct peripheral organs, remains, to a large extent, unclear. GABA_A_R subunit mRNA expression has only been demonstrated in a few peripheral organs of the rat (Akinci and Schofield, 1999). Here, GABA_A_R subunit mRNA expression in mouse bladder, heart, kidney, liver, lung, and stomach was investigated, as a surrogate indicator of potential GABA_A_R subtypes expressed by these organs, with brain tissue used as a positive control. RT-PCR analyses revealed that GABA_A_R subunits are expressed within all the organs investigated and that the expression of these receptors within peripheral organs is subunit-specific (**Figure 1**; *N* = 3 animals). The following GABA_A_R subunit mRNA expression profiles were detectable in the following organs: (1) Stomach (α1–5, β2 and 3, γ2 and 3, and ε); (2) lung (α1 and 3–5, β1, γ3, and ε); (3) bladder (α1 and 3–5, β1–3, δ, and ε); (4) kidney (α1–5, β1–3, γ1 and 3, δ, and ε); (5) heart (α1–5, β3, and ε); and (6) liver (α1–5, β1 and 3, δ, and ε). Notably, for the ε subunit, the sizes of the amplicons in peripheral organs were noticeably different to that of brain. This is most likely indicative of the expression of different splice variants known to exist and potentially code for truncated protein (Wilke et al., 1997). Therefore, the functional relevance of this subunit in peripheral organ systems is debatable.

In summary, the organs which expressed the majority of GABA_A_R subunits were the stomach (10 different subunits detected) and the kidney (12 different subunits detected). In contrast, the lung (six different subunits) and the heart (five different subunits) expressed significantly fewer numbers of different GABA_A_R subunits.

### GABA_A_R Subunit Protein Expression within Specific Peripheral Organs

**FIGURE 2:**
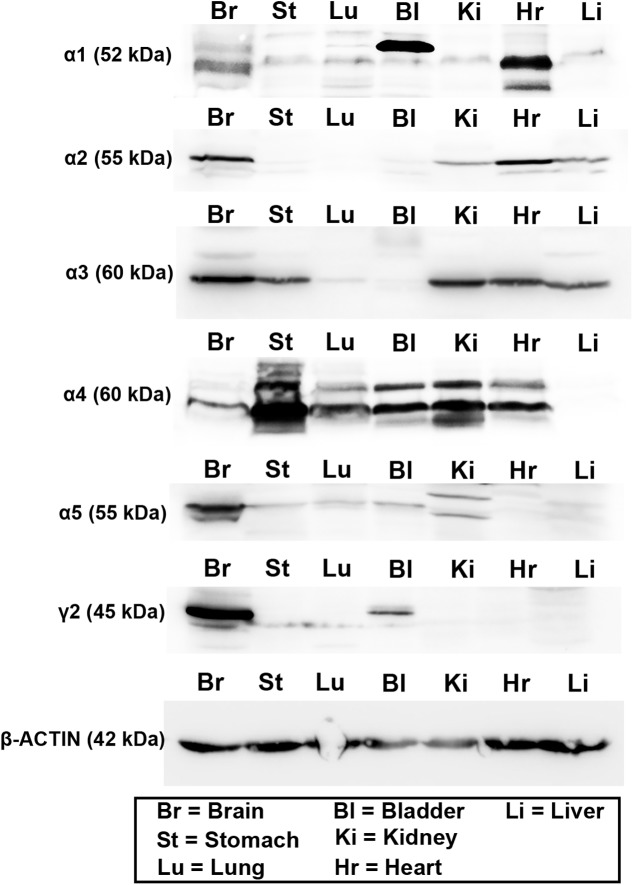
Protein expression of the major GABA_A_R α and γ subunits within various organs of the mouse. Representative western blots for GABA_A_R α1–5 and γ2 subunits using tissue homogenates from whole brain (Br), stomach (St), lung (Lu), bladder (Bl), kidney (Ki), heart (Hr), and liver (Li) obtained from adult male C57BL/6 mice. Western blots for the different subunits in the peripheral organs corresponded to the sizes of those obtained from the brain. Note that blots for actin were run on separate gels due to the similar molecular weight of this protein compared to GABA_A_R subunits. *N* = 3 animals.

The expression of these subunits was then explored at the protein level, using western blotting. This analysis was limited by the availability of antisera which work with this technique, and whose specificity has already been verified in previous studies. As a result, we therefore were unable to investigate all subunits explored at the mRNA level. Therefore, given their known physiological and pharmacological relevance (Rudolph and Knoflach, 2011), we focused only on the α1–5 and γ2 subunits. Representative western blot images are presented in **Figure 2**. Intense bands for all these subunits were detectable in protein homogenates from brain tissue, at the appropriate molecular weight, indicating the specificity of the antisera used. For the α1 subunit, intense bands were detectable in protein homogenates from bladder and heart tissue, with weaker, though specific bands evident in stomach, lung, kidney, and liver homogenates. For the α2 subunit, intense bands were detectable in heart, with weaker expression in kidney and liver. For the α3 subunit, intense bands were detectable in stomach, kidney, heart, and liver. For the α4 subunit, intense bands were detectable in all peripheral organs apart from the liver. For the α5 subunit, intense bands were detectable in kidney and bladder, with weaker expression evident in stomach, lung and liver. Finally, for the γ2 subunit, strong expression was detectable only in bladder, with weaker expression evident in stomach and lung, which could indicate the antibody recognizing an alternative splice variant of the protein. It is notable that bands from bladder samples were of different sizes to that of the brain. Qualitatively, it is evident that GABA_A_R subunit protein varies greatly across organs and subunits, when compared against the total amount of protein per organ. This could be due to either a limited expression of a subunit within an organ *per se*, or strong expression, but within very restricted tissue compartments of an individual organ.

### Changes in the mRNA Expression of KCC2, NKCC1, and NKCC2 with Development

**FIGURE 3:**
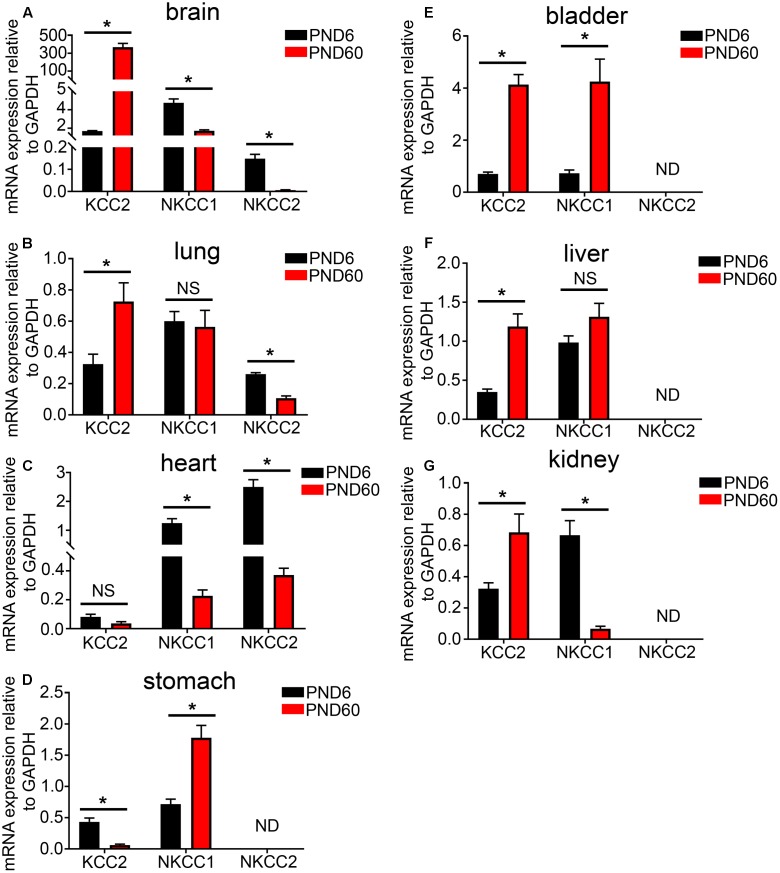
Developmental changes in the mRNA expression level of the KCC2, NKCC1, and NKCC2 chloride ion transporters within various peripheral organs. Quantification of the mRNA expression levels of the potassium-chloride transporter member 5 (KCC2), Na–K–Cl cotransporter 1 (NKCC1), and Na–K–Cl cotransporter 2 (NKCC2) in tissue from mice aged postnatal day 6 (PND 6) and adulthood (PND 60), using qPCR. Within the **(A)** brain, **(B)** lung, and **(C)** heart, a significant increase in KCC2 was detected and a decrease in the NKCCs mRNA levels as the mice age. However, within the **(D)** stomach, the contrary is true. In addition, there is an increase in both KCC2 and NKCCs mRNA expression levels from PND 6 to 60 within the mouse bladder **(E)** and liver **(F)**. Within the mouse **(G)** kidney, KCC2 mRNA is expressed at very low levels in compare to the NKCCs and aging induces a decrease in the expression of NKCCs. Data represent mean ± SEM; *N* = 5 animals. NS, not significant; ND, not detected. ^∗^*P* < 0.05, Student’s unpaired *t*-test.

Using real time qPCR, we compared the relative mRNA expression of KCC2, NKCC1, and NKCC2 transporters within various organ systems of mice aged PND 6 and 60. Within the mouse brain, as expected, we observed a steep developmental upregulation in the mRNA expression of KCC2 and downregulation of NKCC1 and NKCC2 (**Figure 3A**). A similar trend was evident in tissue from lung (**Figure 3B**) and heart (**Figure 3C**). This suggests that similar to the brain, activation of GABA_A_Rs within these peripheral organs will have a depolarizing effect during early postnatal development and a hyperpolarizing effect during adulthood.

In stark contrast, within the mouse stomach, aging resulted in a significant decrease in the expression of the KCC2 and an increase in the mRNA expression of NKCC1 (**Figure 3D**). This finding is in agreement with the current dogma that GABA has a depolarizing and thus excitatory effect within the GI tract and the ENS. There was a detectable increase in both KCC2 and NKCC1 mRNA expression levels within the mouse bladder (**Figure 3E**) and liver (**Figure 3F**).

Within the mouse kidney, NKCC1 and NKCC2 are the predominant Cl ion transporters (**Figure 3G**). This suggests an excitatory effect for GABA within the mouse kidney. Collectively, the data suggest that, in adulthood, cells from different organs may produce contrasting chloride gradients across their membranes. When associated with GABA_A_Rs, the implication is that the activation of a specific GABA_A_R subtype may opposite effects on cellular excitation, that is, either hyperpolarization or depolarization, in different organs.

### ELS-Induced Alterations in the mRNA Expression of GABA_A_R Subunits within Specific Peripheral Organs

Psychosocial stress, experienced either in adulthood or during development, alters the expression and function of rodent GABA_A_Rs within the CNS, in a receptor subtype and brain region–specific manner (Caldji et al., 2003; Lamy and Beck, 2010; Martisova et al., 2012; Crawford et al., 2013; Liu et al., 2014; Corteen et al., 2015). However, the effect of stress on peripheral GABA_A_Rs is poorly understood. Using a mouse model of ELS that robustly imparts an enduring hyper-stress phenotype throughout adulthood (Gunn et al., 2013), we quantified the corresponding changes in the mRNA expression levels of the major α and γ GABA_A_R subunits within our target organs, using qPCR.

**FIGURE 4:**
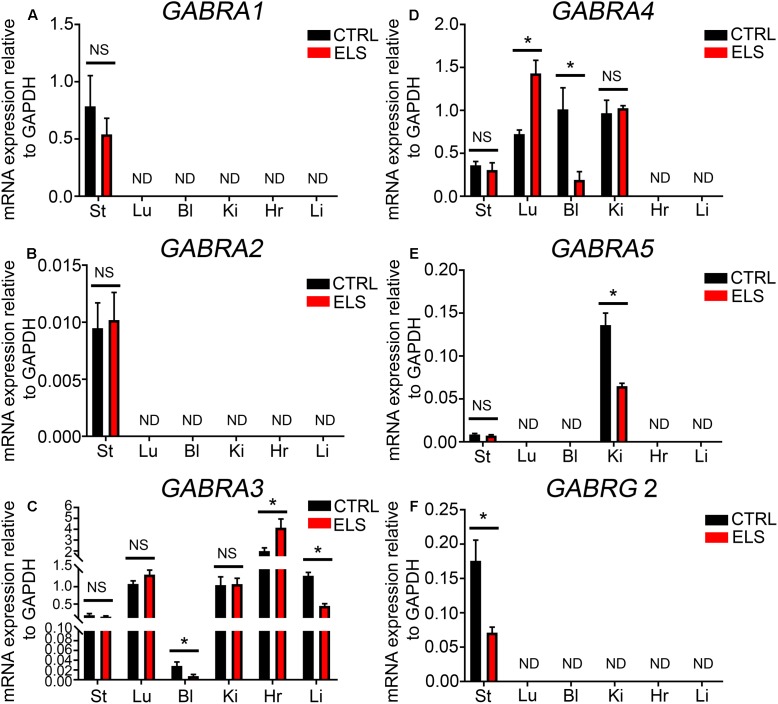
Early life stress (ELS)-induced changes in the level of GABA_A_R subunit mRNA expression within various peripheral organs. Quantification of the ELS-induced changes in the expression levels of the **(A)** α1-GABA_A_R subunit mRNA, **(B)** α2-GABA_A_R subunit mRNA, **(C)** α3-GABA_A_R subunit mRNA, **(D)** α4-GABA_A_R subunit mRNA, **(E)** α5-GABA_A_R subunit mRNA, and **(F)** γ2-GABA_A_R subunit mRNA, within mouse stomach (St), lung (Lu), bladder (Bl), kidney (Ki), heart (Hr), and liver (Li) tissue, relative to the housekeeping gene GAPDH, using qPCR. Note that the mRNA expression of some subunits, relative to GAPDH, was not always detectable (ND) using qPCR. Data represent mean ± SEM; *N* = 5 animals. NS, not significant. Student’s unpaired *t*-test. ^∗^*P* < 0.05.

RT-PCR evidence for the expression of the GABA_A_R α1 subunit was detected within mouse stomach, kidney, and liver (see **Figure 1**). However, when using qPCR and assessed relative to the housekeeping gene Gapdh, the GABA_A_R α1 subunit expression was detectable only within the mouse stomach (**Figure 4A**). Within this organ, there were no significant differences in GABA_A_R α1 subunit mRNA expression between control and ELS subjects (*P* = 0.44, unpaired Student’s *t*-test; *N* = 5 control and ELS animals).

Using qPCR the levels of GABA_A_R α2 subunit expression in comparison to the housekeeping gene Gapdh was negligible in all organs investigated (**Figure 4B**). Within the brain, stress has been shown to significantly increase the expression of the GABA_A_R α3 subunit (Corteen et al., 2014). Here, ELS induced a significant decrease in the mRNA expression of the GABA_A_R α3 subunit within the mouse bladder (*P* = 0.04, unpaired Student’s *t*-test; *N* = 5 control and ELS animals) and liver (*P* < 0.0001, unpaired Student’s *t*-test; *N* = 5 control and ELS animals) (**Figure 4C**). In contrast, within the heart, ELS induced a significant increase in the expression of this GABA_A_R subunit (*P* = 0.03, unpaired Student’s *t*-test; *N* = 5 control and ELS animals) (**Figure 4C**). There were no significant changes in GABA_A_R α3 subunit expression within the stomach, lung, and kidney.

Stress-induced GABA_A_R α4 subunit expression plasticity has yet to be reported within the CNS. Here, ELS induced a significant increase in the expression of the α4 subunit within mouse lung (*P* = 0.0024, unpaired Student’s *t*-test; *N* = 5 control and ELS animals) (**Figure 4D**). In stark contrast, ELS induced a significant decrease in the expression of the GABA_A_R α4 within the mouse bladder (*P* = 0.01, unpaired Student’s *t*-test; *N* = 5 control and ELS animals). However, the expression of the α4 subunit within the mouse stomach and kidney was not significantly altered as a result of ELS.

Using qPCR, GABA_A_R α5 subunit was detectable within the mouse kidney but not in the lung, bladder, stomach, heart, or liver (**Figure 4E**). Within the kidney, ELS resulted in a significant decrease in the expression of the GABA_A_R α5 subunit (*P* = 0.0024, unpaired Student’s *t*-test; *N* = 5 control and ELS animals).

Using qPCR, the GABA_A_R γ2 subunit mRNA expression was only detectable within the mouse stomach and not the lung, bladder, kidney, heart, or liver (**Figure 4F**). Within the stomach, ELS induced a significant decrease in the expression of the GABA_A_R γ2 subunit (*P* = 0.01, unpaired Student’s *t*-test; *N* = 5 control and ELS animals). All these data are summarized in **Table 1**.

Collectively, these data demonstrate that early life environment has an enduring impact on the expression levels of GABA_A_R subunits, within various organs, in adulthood. Furthermore, the trajectory of ELS-induced GABA_A_R subunit expression plasticity is organ-specific.

### ELS Alters the mRNA Expression of KCC2, NKCC1, and NKCC2 within Specific Peripheral Organs in Adulthood

**FIGURE 5:**
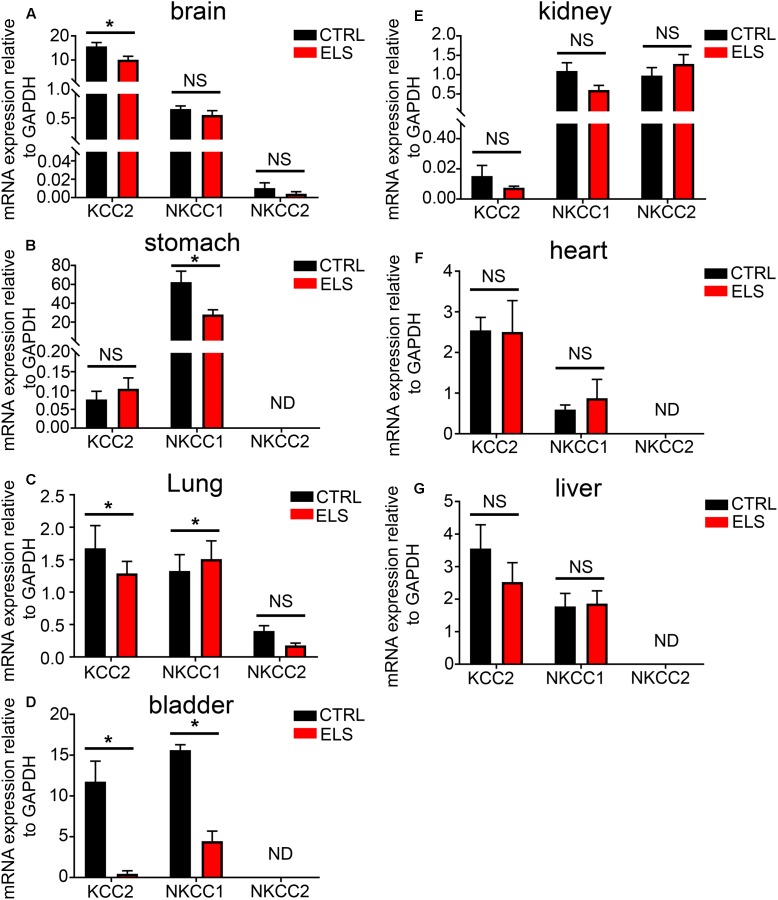
Early life stress (ELS)-induced changes in the level of the potassium-chloride transporter member 5 (KCC2), Na–K–Cl cotransporter 1 (NKCC1), and Na–K–Cl cotransporter 2 (NKCC2) within various peripheral organs. Quantification of the mRNA expression levels of the KCC2, NKCC1, and NKCC2 in **(A)** brain, **(B)** stomach, **(C)** lung, **(D)** bladder, **(E)** kidney, **(F)** heart, and **(G)** liver tissue from adult mice (PND 60) exposed to ELS. Data represent mean ± SEM; *N* = 5 animals. NS, not significant. Student’s unpaired *t*-test. Note that the ELS induced significant changes in the mRNA expression of ion transporters specifically within the brain, stomach, lung, and bladder of the mouse. ^∗^*P* < 0.05.

Given the importance of KCC2, NKCC1, and NKCC2 expression to GABA_A_R function, and the impact that ELS had on GABA_A_R expression in adulthood, we investigated whether ELS also altered the adulthood phenotype of these transporters in peripheral organs. ELS induced a significant decrease in the mRNA expression of KCC2 in the brain. No significant differences were detected for NKCC1 and NKCC2 (**Figure 5A**). Within the stomach, ELS significantly decreased the expression of NKCC1 (**Figure 5B**). ELS significantly decreased the expression of KCC2, and increased the expression of NKCC1 in lung (**Figure 5C**). ELS had a profound effect on the expression of these transporters in the bladder by dramatically decreasing the expression of both KCC2 and NKCC1 (**Figure 5D**). There were no significant effects of ELS on the expression of these transporters in kidney, heart, and liver (**Figures 5E–G**, respectively). Thus, early life environment not only engages peripheral GABA_A_R but also the transporters that determine the gradients of ions which permeate these ion channels.

## Discussion

The current study advances our understanding of the rich diversity of GABA_A_R subunit expression patterns beyond the CNS, by demonstrating potentially unique receptor subtype profiles within various organ systems. Furthermore, the ontogenic expression profiles of the chloride transporters that determine whether the activation of such GABA_A_Rs leads to cellular activation or inhibition, are also organ specific. Finally, we provide evidence that early life psychological stress directly engages the transcriptional machinery of the peripheral GABA_A_R system, in an organ- and subunit-specific manner.

### Technical Considerations

For most of this study, mRNA was used as a surrogate measure of the active expression of specific subunits by individual organs. The obvious caveat is that mRNA expression on its own does not unequivocally prove the expression of the functional protein product. However, initial GABA_A_R mRNA screens within the brain (Wisden et al., 1992) have proved to be indispensable in the design of future analyses within the CNS, and thus the overall advancement of this field within this organ. Nevertheless, to provide a translational insight to these expression profiles, we undertook a protein analysis of the major GABA_A_R subunits in these peripheral organs. Unfortunately, our analyses were limited by the availability of GABA_A_R subunit antisera which have been confirmed to exhibit subunit specificity, as well as perform in western blots as opposed to other protein assays such as immunohistochemistry. There was general agreement between the datasets with subunit protein being detected *only* if mRNA expression was evident within a particular organ. The only outlier was the γ2 subunit in the bladder. We did not detect any mRNA for this subunit, but did obtain a band within this organ. However, the size of the band observed in the bladder was different to that of the brain. There were of course some organs that expressed subunit mRNA and not protein, suggesting that for these subunits, their expression may be limited to the transcriptional level.

A technical confound was the discrepancies in subunit mRNA expression patterns obtained in peripheral organs when using the RT-PCR and qPCR methods. Essentially, the qPCR method failed to detect some subunits which were revealed using RT-PCR, in specific peripheral organs. A possible explanation is the differences in primer sequences or their amplification efficiencies. We therefore performed RT-PCR using our qPCR primers and reliably detected amplicons for these subunits in these organs (**Supplementary Figure 1**). Therefore, the most likely explanation relates to the *relative expression* of the subunit that is reported with qPCR, when normalized against a housekeeping gene. If the subunit is expressed in discrete cells of the entire organ, or in limited amounts compared to the housekeeping gene, then it is likely that such signal will be difficult to detect, when normalized to that of an abundant housekeeping gene found throughout the organ. Therefore, the suggested future, high resolution molecular and histological analyses in individual organs (see below) will be required to place the role of individual subunits within the wider context of organ function. Nevertheless, the data presented here provide a platform for guiding further analyses on the candidate subunits which are most likely to be relevant in terms of GABA_A_R-mediated regulation of these major organs.

Since whole tissue homogenates were used for RNA extraction in this study, we were unable to identify which particular cell types, within a specific organ, express distinct GABA_A_R subunits. Such information is particularly important for predicting the potential functional consequences of GABA_A_R activation on overall organ function. However, technical difficulties relating to the separation of various cell types within the tissue and extracting mRNA from such cell types individually, precluded such analyses. Therefore, future immunohistochemical studies aimed at investigating the cellular and subcellular expression pattern of GABA_A_R subunits within peripheral organs are essential, informed by these data.

### Putative GABA_A_R Subtypes across Various Organs

Our data largely correspond to previous reports on the repertoire of GABA_A_R subunits expressed in the peripheral organs of mouse (Tyagi et al., 2007), and the lungs of rat (Jin et al., 2008) and human (Mizuta et al., 2008; Zhang et al., 2013). The major discrepancy between this report and a related study in mouse is that they (Tyagi et al., 2007) did not detect α1, β1, β3, and γ1 subunit expression in any peripheral organs even though these were detected in rat (Akinci and Schofield, 1999). Given the time-frame between these studies, a parsimonious explanation could be differences in reagents and technical protocols.

The current data reveal that, unlike the CNS which expresses all known GABA_A_R subunits, mouse peripheral organs express only subsets of subunit combinations. Although co-expression does not necessarily imply co-assembly, these data do invite cautious extrapolations of potential receptor subtypes, within individual organ systems, compared to the brain. Firstly, within the brain, the α1 and 2 subunits are considered to be the most widely expressed of all α subunits. However, the current data indicate that the expression of these two subunits is rather limited across all the organs surveyed. Instead, the α3–5 subunits are more widespread, being detectable in all organs investigated. Once again, this is in contrast to the brain where these particular α subunits are enriched within specific regions (Hortnagl et al., 2013).

Another striking difference between the brain and peripheral GABA_A_R subunit expression profiles is the restricted distribution of the γ2 subunit in the organs surveyed; indeed, transcripts for this subunit were detected only in stomach. In contrast, the ε subunit was expressed throughout all organs. An important caveat is that amplicons for the ε subunit in peripheral organs were notably of a different size compared to brain. Since a number of ε splice variants, which putatively encode for truncated proteins, are thought to be expressed particularly within peripheral organs (Wilke et al., 1997), the role for subunits auxiliary to α/β counterparts may be occupied by γ/δ subunits rather than ε. No specific parcellation of individual β subunits, within specific organs was noticeable. In this understandably limited set of organs, the major peripheral receptor subtypes are most likely to be combinations of: (α) α3–5 > α1 > α2; (β) β3 > β1 > β2; (γ/δ/ε) ε > δ–γ3 > γ1–2. If the conventional CNS GABA_A_R subunit stoichiometry of 2α/2β/1γ–δ–ε holds true for the PNS as well, then these data suggest that the major peripheral GABA_A_R subtypes are likely to be composed of α3–5/β3/ε subunits.

These potential GABA_A_R subtypes have implications for drug design if off-target effects are to be avoided. This is important given the growing evidence of different GABA_A_R subtypes having marked contributions to the functioning of various peripheral organs. Many clinically available GABA_A_R-targeting drugs for CNS disorders require the presence of the γ2 subunit. The current study highlights the predominance of the ε subunit over the γ2 within the periphery. This could be exploited in future drug design strategies in order to minimize unwanted effects when targeting either CNS or PNS disorders with GABA_A_R-based therapies.

Emerging evidence indicates the widespread effects of GABA_A_Rs on the native functioning and pathologies of various organs. For example, GABA_A_Rs have been shown to have a pronounced effect on various facets of lung function, ranging from inflammation (Yocum et al., 2017), asthma (Forkuo et al., 2017), lung cancer (Liu et al., 2016) to airway smooth muscle contractility (Gallos et al., 2015). However, within other peripheral organs such as kidney, relatively less is known about the role of GABA–GABA_A_R system. Importantly, many of the actions arise from a direct effect on the organs, rather than through centrally mediated mechanisms. This could therefore represent opportunities for the repurposing and use in peripheral disorders, of discarded GABA_A_R subtype-specific ligands because of their inability to cross the blood–brain barrier.

To achieve this, it is essential for dedicated studies on individual organ systems to identify the roles of individual receptor subtypes in the functioning of specific organs. The availability of GABA_A_R transgenic mouse models, already generated for CNS studies (Crestani and Rudolph, 2015), will be indispensable for this purpose. However, the data also suggest the need for additional models targeting other subunits, such as epsilon, which, in terms of CNS GABA_A_R studies may have not been warranted given its restricted expression in the brain. Indeed, given the ubiquity of the epsilon subunit in the periphery demonstrated by these data, it is reasonable to predict a significant functional role, thus placing it as a prime target for future such studies.

### Plasticity of the Peripheral GABA_A_R System

The results of this study suggest that the function of the peripheral GABA_A_R subtypes, and associated transporters, are likely to change dynamically, at least with age, as well as with early life experience. The effect of the GABA–GABA_A_R system on cellular excitability, namely hyperpolarization or depolarization, is determined by the directional flow of chloride ions, down a concentration gradient upon agonist binding. The expression levels of chloride transporters are thus integral to GABA_A_R-mediated cellular excitability as this determines the relative chloride ion concentrations across the plasma membranes of the cell system of interest. This is elegantly illustrated in the developing CNS where an initial excitatory role for GABA_A_Rs during development is transformed to that of neuronal inhibition in adulthood, due to the increased expression of the KCC2 with age. Such ontogenic expression profiles of associated chloride transporters are poorly understood in peripheral organs and the PNS. The current study indicates that the developmental expression profiles of the major chloride transporters that determine cellular chloride gradients, namely the NKCC1, NKCC2, and KCC2, change dynamically during development, in an organ-specific manner. The implication is that an individual GABA_A_R subtype could have contrasting effects on cellular excitability depending on the individual organ.

A striking finding of this study was that prior experience of ELS had a robust effect on expression of GABA_A_R subunit and ion transporter levels in adulthood, although in an organ-specific manner. Convergent lines of evidence indicate that various forms of stress robustly engage brain GABA–GABA_A_R systems (Maguire, 2014). Importantly, early life adversity is known to have a profound effect on multiple organ systems, not only the nervous system. Indeed, ELS confers an enduring vulnerability to developing a range of illnesses later on in life, such as mental illnesses, cardiovascular and metabolic disorders (Shonkoff et al., 2009; Miller et al., 2011). With an ever aging population, there is a growing urgency to identify the underlying biological mechanisms through which experiences in childhood engage such a wide variety of body systems, resulting in a multitude of pathologies in later life. A considerable body of evidence indicates that brain disorders in adulthood due to prior ELS arise from epigenetic, morphological, and physiological changes in various CNS pathways (McEwen et al., 2015). However, the role of the PNS in mediating the negative effects of ELS in various peripheral organs is less well understood. To the best of our knowledge, we provide the first demonstration that ELS engages different branches of the PNS, in an interaction that involves specific organs and GABA_A_R subunits. A tantalizing proposition is that such ELS-induced GABA_A_R expression phenotypes contribute to changes in organ function later on in life. Functional verification of this proposition could provide unique molecular targets for addressing such medical conditions.

Conceptually, the question arises as to how one’s environment, or experience thereof, could alter the activity of different GABA_A_R genes, divergently in various organs. Since ELS has been shown to alter gene activity epigenetically within the CNS and such epigenetic alterations result directly in changes at transcriptional levels (Anacker et al., 2014), the questions emerges as to whether the ELS-induced changes in the mRNA expression of GABA_A_R subunits within peripheral organs are due to epigenetic modifications. Hence, the data in this study could provide a platform for further epigenetic studies aimed at identifying drug targets which may counteract the negative impacts of ELS on major peripheral organs via modification of the peripheral GABA–GABA_A_R system. Furthermore, intracellular chloride concentration has been shown to influence the subunit composition of GABA_A_R subtypes, in particular α3-GABA_A_R (Succol et al., 2012). Coincidently, we have previously demonstrated repeated stress in adulthood increases the expression of brain α3-GABA_A_Rs (Corteen et al., 2015). The current data indicate that ELS alters the expression of membrane transporters integral to regulating intracellular chloride concentration. Therefore, some of the ELS induced changes in the expression of GABA_A_R subunits demonstrated in this study, or in previous reports (Corteen et al., 2015), could be due to alterations in chloride gradients arising from the associated changes in the expression of the relevant membrane transporters. If so, and given the ubiquity of other cellular processes underpinned by such ionic gradients, such ELS-induced changes have the potential to impart significant changes on a variety of cellular processes, which could underlie the associated pathologies.

In summary, the study provides an outline of the diversity of GABA_A_R subunits, expressed at the mRNA level, in major peripheral organs. These data provide a platform for future functional analyses of the contribution of the GABA–GABA_A_R system to the health, and associated diseases of these particular organ systems.

## Author Contributions

JS and MS designed the research. EE, AG, JS, and MS performed the experiments. EE, AG, and MS analyzed the data. JS and MS wrote the manuscript.

## Conflict of Interest Statement

The authors declare that the research was conducted in the absence of any commercial or financial relationships that could be construed as a potential conflict of interest. The reviewer TJ and the handling editor declared their shared affiliation.

## Supplementary Material

FIGURE S1 Performance of qPCR primers using the RT-PCR methods. Representative gel electrophoresis image of mRNA transcripts for various GABA_A_R subunits, demonstrating the performance of qPCR primers using the RT-PCR method, on homogenates from the whole brain (Br), stomach (St), lung (Lu), bladder (Bl), kidney (Ki), heart (Hr), and liver (Li) obtained from adult male C57BL/6 mice.

## Abbreviations

CNS: central nervous system
ELS: early life stress
ENS: enteric nervous system
GABA: gamma aminobutyric acid
KCC2: potassium-chloride transporter member 5
NKCC1: Na–K–Cl cotransporter 1
NKCC2: Na–K–Cl cotransporter 2
PND: postnatal day
PNS: peripheral nervous system
