# Supplementary figures and images for: Molecular Characterization of GABA-A Receptor Subunit Diversity within Major Peripheral Organs and Their Plasticity in Response to Early Life Psychosocial Stress
Source: Front Mol Neurosci. 2018 Feb 6;11:18. doi: 10.3389/fnmol.2018.00018 (PMC5807923; doi:10.3389/fnmol.2018.00018)

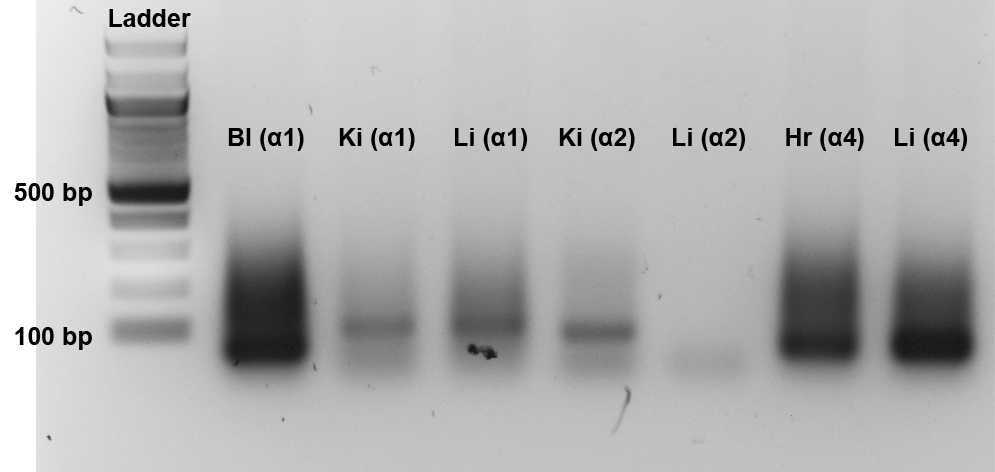

Supplement: FIGURE S1 — Performance of qPCR primers using the RT-PCR methods. Representative gel electrophoresis image of mRNA transcripts for various GABAAR subunits, demonstrating the performance of qPCR primers using the RT-PCR method, on homogenates from the whole brain (Br), stomach (St), lung (Lu), bladder (Bl), kidney (Ki), heart (Hr), and liver (Li) obtained from adult male C57BL/6 mice. [file Image_1.TIFF]
